# Neurosarcoidosis Presenting With Cerebellar Hemorrhage and Spinal Cord Involvement: A Case Report

**DOI:** 10.7759/cureus.102118

**Published:** 2026-01-22

**Authors:** Seyed Mostafa Razavi, Emily Storm, Karan K Topiwala

**Affiliations:** 1 Department of Neurology, University of South Dakota Sanford School of Medicine, Sioux Falls, USA; 2 Department of Pathology, University of South Dakota Sanford School of Medicine, Sioux Falls, USA

**Keywords:** cerebellar hemorrhage, intracranial hemorrhage, neurosarcoidosis, sarcoidosis, spinal involvement

## Abstract

Neurosarcoidosis is a rare manifestation of sarcoidosis, a systemic granulomatous disease. It presents a diagnostic challenge due to its varied clinical and radiologic features.

We describe a 37-year-old male with biopsy-proven pulmonary sarcoidosis who developed progressive neurological symptoms, including dizziness, imbalance, diplopia, and lower extremity weakness. MRI revealed diffuse leptomeningeal enhancement, cervical spinal cord lesions, and left cerebellar hemorrhage with surrounding vasogenic edema, a relatively rare finding in neurosarcoidosis. Cerebrospinal fluid (CSF) analysis showed inflammatory changes, and mediastinal lymph node biopsy confirmed non-necrotizing granulomas. The patient was treated with high-dose corticosteroids followed by immunosuppressive therapy, leading to gradual improvement in gait and strength.

This case highlights the importance of recognizing neurosarcoidosis as a potential cause of central nervous system hemorrhage. Cerebrovascular involvement, particularly intracerebral hemorrhage, is underrecognized but can have serious consequences, making early diagnosis and aggressive immunosuppressive therapy critical in preventing long-term neurological deficits.

## Introduction

Sarcoidosis is an idiopathic, granulomatous disease most commonly affecting the lungs but can potentially involve other organ systems, including the central nervous system (CNS). Neurosarcoidosis (NS) occurs in approximately 5-15% of sarcoidosis cases and is often underrecognized due to its diverse clinical manifestations, including cranial neuropathies, leptomeningitis, and myelopathy [1]. The pathophysiology of NS remains poorly understood but is thought to involve perivascular and leptomeningeal inflammation leading to granuloma formation, which may predispose to vascular injury and cerebrovascular complications [2].

The clinical presentation of NS varies widely, ranging from cranial neuropathies to myelopathy and cerebrovascular complications. While ischemic stroke is a reported manifestation of neurosarcoidosis, hemorrhagic presentations are exceedingly rare, with only a small number of cases reported in the literature, and may mimic other vascular or structural lesions [3]. MRI is a crucial diagnostic tool in neurosarcoidosis, often revealing leptomeningeal enhancement, parenchymal lesions, and spinal cord involvement, as demonstrated in our patient. Diagnosis is further supported by cerebrospinal fluid (CSF) analysis, biopsy findings, and exclusion of other etiologies [4].

Here, we report a case of probable NS with biopsy-proven pulmonary sarcoidosis presenting with progressive neurological symptoms and complicated by a cerebellar hemorrhage, highlighting the importance of considering NS in patients with unexplained CNS hemorrhage.

## Case presentation

Patient information

A 37-year-old male with past medical history of gastroesophageal reflux disease, obesity, and pulmonary sarcoidosis that was diagnosed two months earlier, presented with subacute, progressively worsening neurological symptoms over several weeks. He did not have any prior history of hypertension, known coagulopathy, stroke, multiple sclerosis, or other autoimmune diseases, aside from sarcoidosis. His family history was notable for an uncle with sarcoidosis.

Initial clinical course

**Figure 1:**
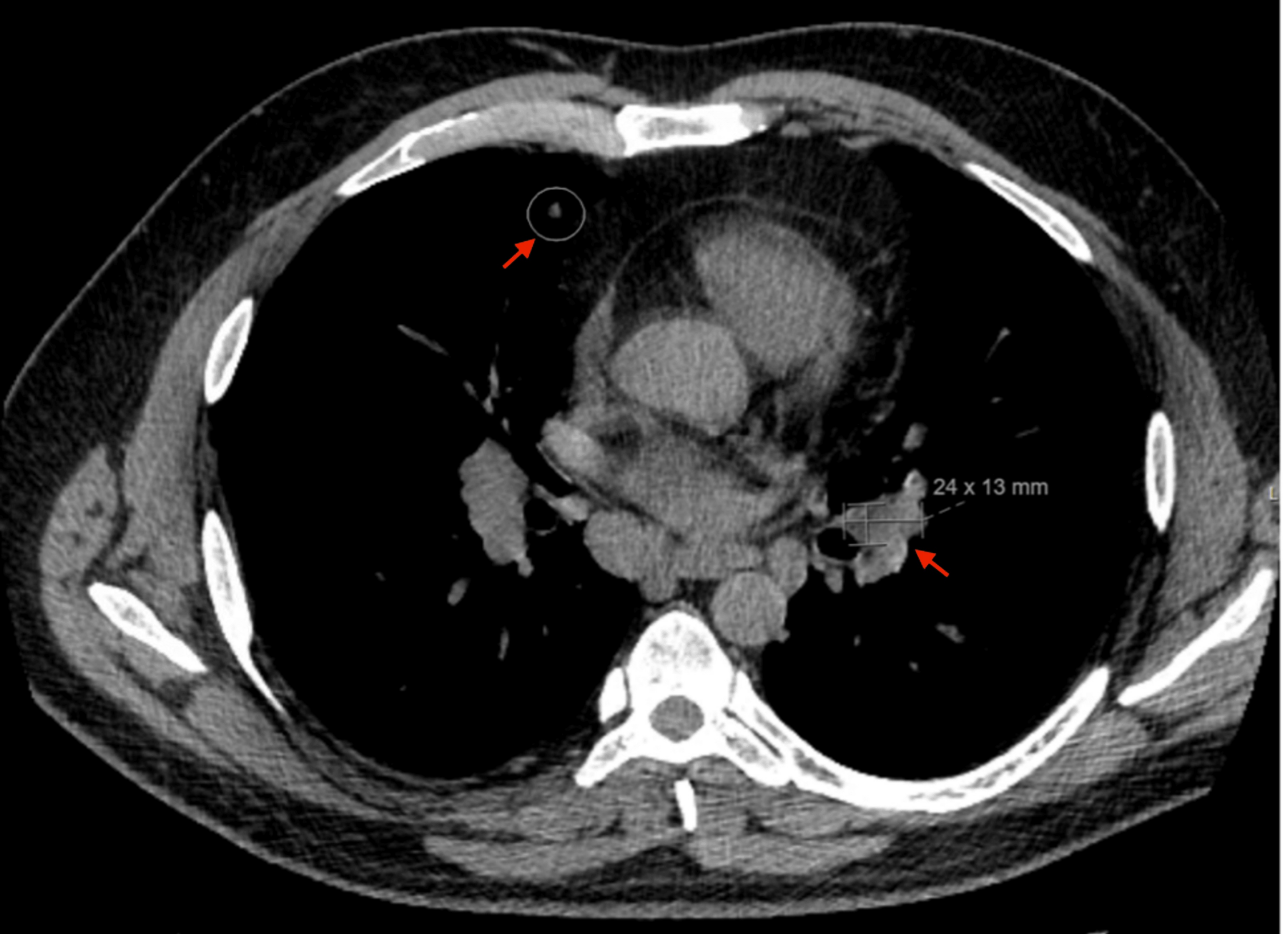
CT scan of the chest showing mediastinal and bilateral hilar lymphadenopathy in addition to scattered pulmonary micronodules

**Figure 2:**
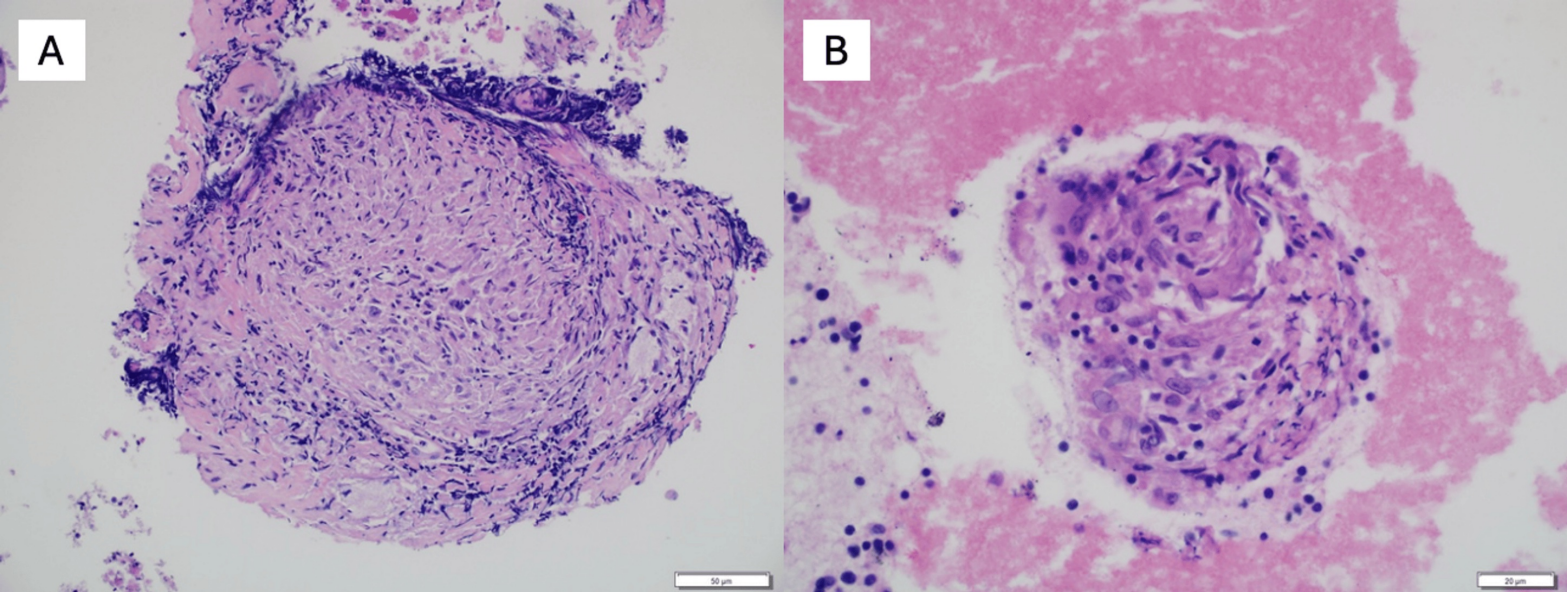
Bronchial ultrasound-guided fine-needle aspiration (EBUS- FNA) of mediastinal lymph nodes demonstrating nonnecrotizing granulomatous inflammation (A) Sample from station 4R lymph node (20× objective) shows well-formed granulomas with chronic inflammatory infiltrate composed of histiocytes and scattered multinucleated giant cells; (B) Sample from station 7 lymph node (40× objective) highlights granuloma architecture in greater detail. Images were acquired using cellSens Standard software (version 1.6) on an Olympus BX46F microscope with DP72 digital camera using 20× and 40× objectives (Olympus Corporation, Tokyo, Japan)

The patient first sought medical attention for a chronic cough, intermittent nausea, and vomiting. Pulmonary function tests revealed a restrictive pattern with reduced forced vital capacity, total lung capacity, and diffusing capacity for carbon monoxide. A high-resolution CT scan of the chest demonstrated mediastinal and bilateral hilar lymphadenopathy with perilymphatic pulmonary micronodules, best appreciated on lung window imaging (Figure 1). A bronchoscopy with endobronchial ultrasound-guided fine-needle aspiration biopsy of mediastinal lymph nodes (stations 4R and 7), performed prior to this hospitalization, confirmed non-necrotizing granulomatous inflammation consistent with sarcoidosis (Figure 2). Grocott's methenamine silver (GMS) stains were negative for fungal organisms, and AFB stains were negative for acid-fast bacilli. Bronchial lavage cytology was negative for malignant cells. At the time, his symptoms were mild, and no systemic therapy was initiated.

Several weeks after the onset of his initial respiratory symptoms, the patient developed dizziness, balance disturbances, blurry vision with diplopia, and bilateral tinnitus. He also reported episodic lower extremity weakness and urinary retention. Neurological examination demonstrated gait instability with impaired balance and lower extremity weakness. Given the patient's history of sarcoidosis, there was concern for NS, which prompted further workup.

Diagnostic workup and treatment

**Figure 3:**
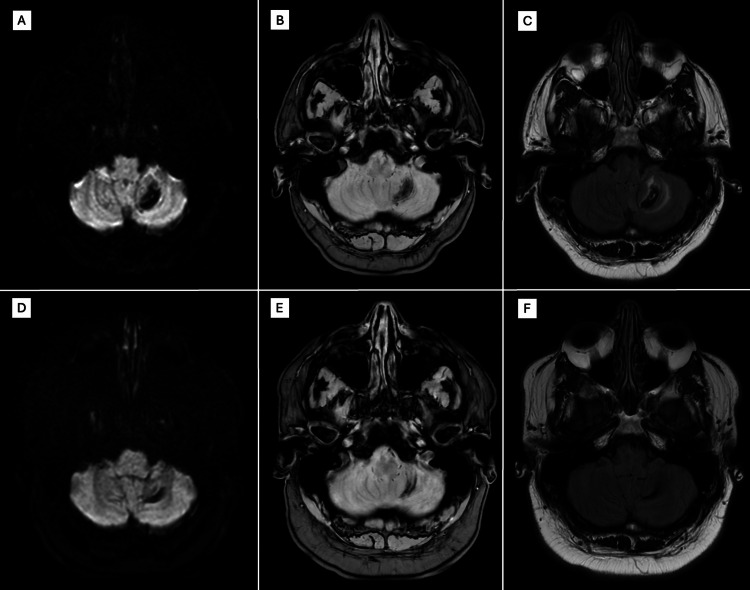
Cerebellar hemorrhagic lesion of neurosarcoidosis Initial diffusion-weighted imaging (DWI) (A), gradient echo sequences (GRE) (B), and fluid-attenuated inversion recovery (FLAIR) (C) brain MRI sequences showed restricted diffusion, blood products, and vasogenic edema around the neurosarcoidosis lesion in the left cerebellar hemisphere. Follow-up brain MRI at six-month follow-up with DWI (D), GRE (E), and FLAIR (F) sequences showed improvement of hemorrhage and resolution of vasogenic edema.

**Figure 4:**
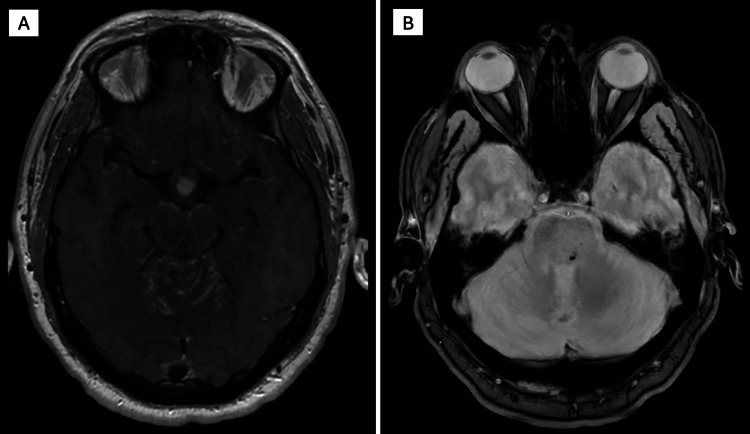
Brain MRI showing hypothalamic/pituitary enhancement and pontine microhemorrhage in neurosarcoidosis (A) Post-contrast T1 brain MRI showing leptomeningeal enhancement and thickening of the hypothalamus and proximal pituitary stalk; (B) MRI gradient echo sequences (GRE) sequence showing posterior medial pontine microhemorrhage.

**Figure 5:**
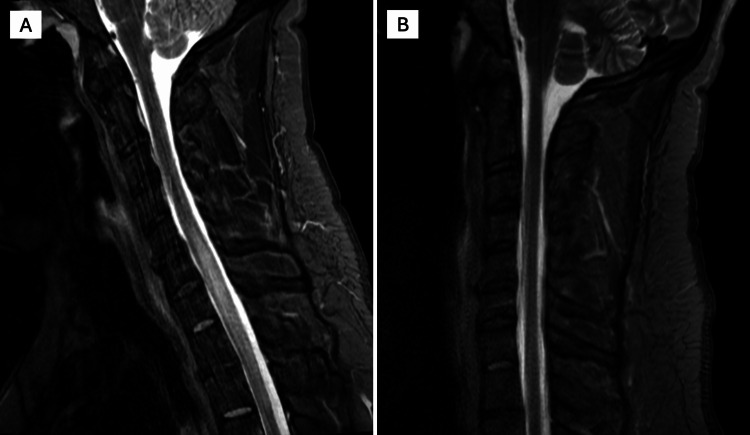
Spinal lesion of neurosarcoidosis (A) MRI of the cervical spine showing T2 hyperintensity in the cervical spinal cord from C5-T2; (B) Repeat cervical MRI at six-month follow-up showing resolution of hyperintensity.

The patient was admitted for worsening lower extremity weakness, urinary retention, and unsteady gait, which were evaluated by the neurology service and raised concern for cerebellar and spinal cord involvement. MRI of the brain revealed a 22 × 12 mm left cerebellar hemorrhage with surrounding vasogenic edema (Figure 3). Leptomeningeal enhancement involved the bilateral cerebral hemispheres, cerebellum (Figure 4A), brainstem, and multiple cranial nerves, including the optic and oculomotor nerves, along with thickening of the hypothalamus and proximal pituitary stalk (Figure 4A). No associated abnormalities were identified on endocrine laboratory evaluation. Punctate foci of microhemorrhage were also noted in the pons (Figure 4B). MRI of the cervical and thoracic spine demonstrated mild expansile T2 hyperintensity from C5-T2, leptomeningeal enhancement extending into the brainstem, cerebellum, and spinal cord, and intramedullary enhancement suggestive of inflammatory or granulomatous disease (Figure 5).

CSF analysis showed an elevated nucleated cell count (244/µL, lymphocyte predominant), elevated protein (234.7 mg/dL), and low glucose (34 mg/dL); bacterial, fungal, and mycobacterial cultures were obtained and showed no growth. Oligoclonal bands were detected (six in CSF vs. four in serum). CSF infectious studies, including AFB smear, fungal culture, and viral PCRs, were negative.

Based on the patient's clinical presentation and diagnostic workup, the diagnosis of probable neurosarcoidosis was made according to the 2018 Neurosarcoidosis Consortium Consensus criteria [2], and high-dose intravenous methylprednisolone 1000 mg/day was initiated for five days, followed by oral prednisone 60 mg daily with a planned gradual taper. A diagnostic cerebral angiogram ruled out vascular malformations or vasculitis. 

The patient was transferred for inpatient rehabilitation, where he underwent intensive physical and occupational therapy. Lower extremity strength improved, though residual balance disturbances persisted. Given the extent of his neurological involvement, long-term immunosuppressive therapy was initiated, including a prednisone taper (starting at 50 mg daily), methotrexate 10 mg weekly initiated at a low dose for tolerability with planned escalation to 15 mg after three months, and infliximab.

Follow-up 

Two months after initiation of therapy, the patient's ambulatory function had improved based on serial neurological examinations and physical therapy assessments, allowing him to ambulate independently without an assistive device, though with residual gait instability. He continued to experience intermittent dizziness and blurry vision, with improvement but incomplete resolution of his diplopia. He remained on a tapering dose of prednisone, methotrexate, and planned infliximab therapy.

At six-month follow-up, repeat MRI demonstrated improvement of the left cerebellar hemorrhage (Figure 3), improvement of previously involved cranial nerves, including the optic and oculomotor nerves, and resolution of prior cervical and thoracic cord signal abnormalities (Figure 5B). Prednisone was discontinued after approximately six months of therapy, and the patient remained on methotrexate and maintenance infliximab infusions every four weeks with good clinical tolerance; neither medication had been initiated at the time of his initial pulmonary sarcoidosis diagnosis, and both were started following the diagnosis of probable neurosarcoidosis. He reported persistent mild balance issues and fatigue that gradually improved over time, for which symptomatic treatment and physical therapy were continued.

## Discussion

Although uncommon, NS represents a serious complication of systemic sarcoidosis, affecting approximately 5-15% of patients with systemic disease [1]. Clinical presentation can be diverse, often leading to diagnostic challenges, particularly in cases with atypical features such as hemorrhagic presentation and diffuse leptomeningeal involvement, as seen in this patient. In this case, a 37-year-old male with known pulmonary sarcoidosis developed progressive neurological symptoms, including dizziness, balance disturbances, visual changes, and lower extremity weakness. These manifestations underscore the variable nature of NS and the importance of maintaining a high index of suspicion in patients with systemic sarcoidosis presenting with new neurological deficits [5].

NS can involve any part of the central or peripheral nervous system; however, simultaneous involvement of cranial nerves, cerebellum, and spinal cord is particularly uncommon. The most common presentation is cranial nerve involvement, with the facial nerve being most frequently affected [2]. However, the patient exhibited a broader range of symptoms, including simultaneous cerebellar and spinal cord involvement, a pattern that is less commonly reported and can be diagnostically challenging because it may mimic demyelinating disease, infectious meningitis, or primary vasculitis.

MRI is a crucial diagnostic tool for NS, as it often reveals leptomeningeal enhancement, parenchymal lesions, or spinal cord involvement [2, 4]. In this case, MRI revealed leptomeningeal enhancement across multiple regions, cervical spinal cord lesions, and a left cerebellar hemorrhage, an atypical finding in NS that increased diagnostic uncertainty by raising concern for alternative vascular or infectious etiologies. Cerebrovascular involvement, although uncommon, has been reported in NS and can lead to both ischemic strokes and hemorrhagic lesions; however, hemorrhagic presentations remain exceedingly rare, with only a limited number of cases reported in the literature [6]. Hemorrhagic manifestations of NS are exceedingly rare, accounting for a small minority of reported cerebrovascular cases, and can be mistaken for other etiologies such as vascular malformations, neoplasms, or hypertensive hemorrhage, all of which were excluded in our patient through comprehensive imaging and diagnostic cerebral angiography [3, 4, 6]. Bathla et al. provided a comprehensive review of cerebrovascular manifestations in NS and emphasized the role of granulomatous vasculitis affecting both small perforating arteries and larger vessels, which may lead to vessel wall fragility and contribute to both infarcts and hemorrhages, as observed in our patient [3]. CSF analysis typically demonstrates elevated protein levels, lymphocytic pleocytosis, and occasionally oligoclonal bands, as observed in our patient; however, this pattern is nonspecific and can further complicate the diagnostic process [2]. 

The definitive diagnosis of sarcoidosis is established through histopathologic confirmation of noncaseating granulomas, which, in our patient, was obtained via mediastinal lymph node biopsy. The diagnostic criteria for NS follow a distinct set of guidelines. In 2018, the Neurosarcoidosis Consortium Consensus Group established diagnostic categories of definite, probable, and possible NS based on clinical features, imaging findings, histopathology, and exclusion of alternative diagnoses [2]. A "definite" diagnosis requires clinical and diagnostic findings consistent with NS, along with pathologic confirmation of granulomatous inflammation within the nervous system and exclusion of alternative diagnoses. The "probable" diagnosis is assigned to cases where there is clinical and diagnostic evidence suggestive of NS, pathologic confirmation of systemic sarcoidosis (e.g., biopsy-proven granulomatous disease in the lungs or lymph nodes, as in our patient), and exclusion of other potential causes. The diagnosis of "possible" NS applies to cases that have a compatible clinical presentation and diagnostic workup but lack histopathologic confirmation of granulomatous disease [7]. Our patient met the criteria for probable NS, as he had the clinical presentation and imaging findings suggestive of NS, along with pathologic confirmation of sarcoidosis in the lungs, but no histopathologic confirmation from the CNS lesions.

The cornerstone of NS treatment is immunosuppression [8]. Corticosteroids are the first-line treatment, often leading to significant clinical improvement. Given the potential for relapse and the adverse effects associated with long-term steroid use, patients are usually switched to steroid-sparing agents such as methotrexate. In refractory cases, tumor necrosis factor-alpha (TNF-α) inhibitors like infliximab have been employed and shown efficacy [3, 8]. Our patient responded well to high-dose corticosteroids and was started on methotrexate and infliximab for long-term immunosuppressive management, given the severity and multifocal involvement of the central nervous system. At six-month follow-up, repeat MRI showed complete resolution of the cerebellar hemorrhage and near-complete resolution of leptomeningeal enhancement, highlighting the effectiveness of early aggressive immunosuppressive therapy.

The clinical course of NS is unpredictable, ranging from self-limiting to progressive disease; in our case, the presentation was severe but demonstrated a favorable response to early aggressive immunosuppressive therapy. Early recognition and prompt initiation of immunosuppressive therapy are crucial in preventing irreversible neurological damage [7]. Regular monitoring with clinical assessments and imaging is essential to evaluate treatment response and adjust the therapy approach. Our case adds to the limited literature on hemorrhagic presentations of NS and underscores that timely diagnosis and comprehensive immunosuppression can lead to significant radiologic improvement and objectively documented functional recovery, as evidenced by improved ambulation on serial neurologic examinations and physical therapy assessments, even in cases with severe CNS involvement.

## Conclusions

This case highlights an uncommon hemorrhagic presentation of NS characterized by simultaneous cerebellar hemorrhage, leptomeningeal disease, and spinal cord involvement, underscoring the diagnostic complexity and clinical significance of this entity. The presence of a cerebellar hemorrhage in our patient represents an extremely rare manifestation of NS and highlights the importance of considering NS in the differential diagnosis of unexplained intracranial hemorrhage, particularly after vascular mimics have been excluded through diagnostic angiography.

This case demonstrates that early recognition and initiation of aggressive, multi-agent immunosuppressive therapy, including corticosteroids, methotrexate, and infliximab, can result in radiologic resolution in severe NS. Neurological recovery was assessed through serial neurologic examinations and documented functional improvement in ambulation. Ongoing clinical and imaging surveillance remains critical to detect relapse and adjust therapy appropriately, typically at intervals of three to six months. Given the rarity of hemorrhagic presentations, additional reports are needed to improve understanding and guide treatment strategies for this unique manifestation.
